# Genome-wide identification of Argonautes in Solanaceae with emphasis on potato

**DOI:** 10.1038/s41598-020-77593-y

**Published:** 2020-11-25

**Authors:** Zhen Liao, Kristian Persson Hodén, Ravi Kumar Singh, Christina Dixelius

**Affiliations:** 1 Department of Plant Biology, Uppsala BioCenter, Linnéan Center for Plant Biology, Swedish University of Agricultural Sciences, P.O. Box 7080, 75007 Uppsala, Sweden; 2 Present Address: Genomics and Bioinformatics Laboratory, University Department of Botany, Magadh University, Bodh Gaya, 824234 India

**Keywords:** Biotic, Plant evolution, Plant molecular biology

## Abstract

Regulatory small RNAs (sRNAs) play important roles in many fundamental processes in plant biology such as development, fertilization and stress responses. The AGO protein family has here a central importance in gene regulation based on their capacity to associate with sRNAs followed by mRNA targeting in a sequence-complementary manner. The present study explored Argonautes (AGOs) in the *Solanaceae* family, with emphasis on potato, *Solanum tuberosum* (*St*). A genome-wide monitoring was performed to provide a deeper insight into gene families, genomic localization, gene structure and expression profile against the potato late blight pathogen *Phytophthora infestans*. Among 15 species in the *Solanaceae* family we found a variation from ten AGOs in *Nicotiana obtusifolia* to 17 in *N*. *tabacum*. Comprehensive analyses of AGO phylogeny revealed duplication of *AGO1*, *AGO10* and *AGO4* paralogs during early radiation of Solanaceae. Fourteen AGOs were identified in potato. Orthologs of *AGO8* and *AGO9* were missing in the potato genome. However, *AGO15* earlier annotated in tomato was identified. StAGO15 differs from the other paralogs having residues of different physico-chemical properties at functionally important amino acid positions. Upon pathogen challenge *StAGO15* was significantly activated and hence may play a prominent role in sRNA-based regulation of potato defense.

## Introduction

Non-coding small RNAs (sRNAs) are ubiquitous components of eukaryotic and prokaryotic gene regulatory processes^1^. Although variation in lengths, biogenesis, functions and targets not least in plants, the unified outcome is restricting the action of target molecules either on transcriptional or post-transcriptional levels. The core part required for this regulatory RNA-based process involves canonical ribonucleases that participate in initiator and effector steps. They are Dicer or DICER-LIKE (DCL) proteins that cleave double-stranded RNA and Argonautes (AGOs), important for the small RNA association and formation of the RNA-induced RNA silencing complexes (RISCs)^2,3^. RNA-dependent RNA polymerase (RDR) is a third enzyme that takes part in amplifying and maintaining the silencing signal in many organisms^4^.

Eukaryotic AGO proteins are characterized by four domains of importance to bind diverse sRNA classes. They are: PAZ (Piwi-Argonaute-Zwille), MID, PIWI, and N-terminal sequences^5^. The four domains have distinct functions to facilitate sRNA loading and activity of RISC^6^. In eukaryotes, the sRNA guide strand is anchored at the PAZ domain via its 3′ end^7^. At the opposise side, MID-PIWI sequences form a binding pocket for the 5′ end^8,9^. In plants, each class of sRNAs display a bias towards certain 5′ nucleotides (nt)^10^, and modulation of RISC turnover rate and possible recycling of AGOs, is channeled via the 3′ sequence attached to the PAZ domain^11^. Apart from Dicer-dependent pathways, AGOs also are involved in Dicer-independent events where for example short hairpin RNA rely on AGO2 for maturation^12^.

There is a wide variation in numbers of *DCL* and *AGO* genes in different organisms^5,13^. *Arabidopsis thaliana* and rice have four and five *DCL*s, respectively, whereas Drosophila has two and *C. elegans* only one *DCL* gene^14^. Similarly, the numbers of AGOs vary greatly in different species. For instance, there is only one *AGO* gene in the fission yeast *S. pombe* but as many as 27 are found in *C. elegans*^15^. Species in the green clade are no exceptions. In the evolution of Viridiplantae, the *AGO* gene family has expanded from three members in green algae^16^ to six in moss, ten in *Arabidopsis,* 19 in rice and 22 in soybean^17,18^. Plant AGO proteins are grouped into three major clades named after phylogenetic analysis and comparison with *Arabidopsis*: AGO1/5/10, AGO2/3/7, and AGO4/6/8/9^19^. These three AGO clades are dated back to the most recent common ancestors of land plants. Thus, the diversification of the AGO family in Viridiplantae is an ancient and most likely a continuous process^20^.

High-throughput sequencing followed by comparative genomics has revealed several gains and losses of AGO encoded genes. In addition, genes not previously annotated are still a source of new information such as reported for *AGO*s in *Nicotiana attenuata*^21^. Species in the *Solanaceae* family have experienced a specific ploidy event after the split with Asterids about 49 million years ago followed by further species divergence^22^. The large genus Solanum diverged from peppers (*Capsicum*) *c.* 19 Million years ago (Mya) whereas the Solanum crops, potato and tomato split rather recently *c*. 8 Mya^23^. Overall, the *Solanaceae* gene families vary in size due to duplications and different gene evolutionary events^24^.

In this study we used all public genomic and transcriptomic resources to clarify the number of *AGO* encoding genes, and their divergence in Solanaceae followed by tests of expression upon filamentous pathogen infection. Extensive gains and losses of *AGO*s have occurred in Nicotiana species compared to Solanum species. The AGO4 clade which harbors a specific Solanaceae sub-clade, AGO15, with novel sequence and structural features received our attention. StAGO15 was activated by filamentous pathogen challenge suggesting an important role in the immune responses of potato. Details of its sRNA binding and function under biotic stress remains to be elucidated.

## Results

### Extensive gains and losses of *AGO*s in Solanaceous genomes

**Figure 1:**
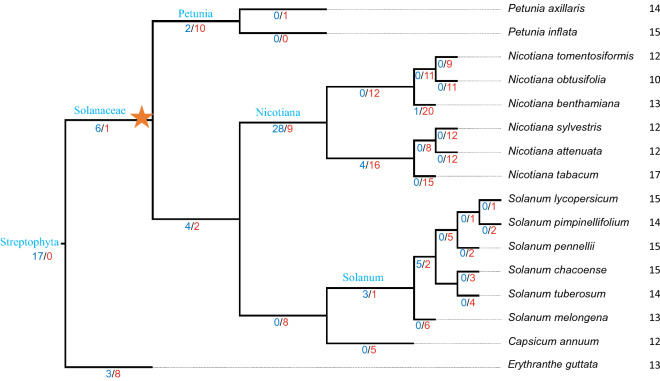
Evolutionary events of the Argonaute (*AGO*) family in Solanceous genomes. The values on branches correspond to the number of gene gains (blue) and losses (red). Right panel showing the total number of *AGO* genes in the genome of each species. ★ indicates a whole genome triplication event.

We searched full length AGO sequences in potato and related genomes of Solanaceae species to generate an overview picture of the number of AGOs in the *Solanaceae* family and their evolutionary history. To generate confidence over number of gene gain and loss events, the reconciliation of the gene trees was repeated three times using three different species as out-groups *Arabidopsis*, *Vitis vinifera* and *Erythranthe guttata*. We found six *AGO* duplication events prior the split between Petunia and the other Solanaceae linages (Fig. 1; Supplementary Fig. S1). After the divergence of Petunia, the ancestral line experienced four duplication and two loss events followed by speciation processes leading to Nicotiana and Solanum lineages. The *AGO* family in the ancestral Nicotiana lineage has experienced extensive changes. Based on the six Nicotiana species analyzed, 33 duplication and 59 loss events had occurred prior further speciation. Processes involving gene losses have continued within each species. Maximum losses have occurred in *N. benthamiana* (20) followed by *N. tabacum* (15). Compared to Nicotiana, the expansion of the *AGO* family is less variable in the Solanum genus. Overall, the number of AGOs in the Solanaceae species analyzed varies from ten in *N*. *obtusifolia* to 17 in *N*. *tabacum*.

### Potato has 14 Argonaute encoding genes

The search for homologous *AGO* sequences in the *Solanaceae *family generated fourteen full length *AGO* genes recovered from the *S. tuberosum* (potato) genome (*StAGOs*). PAZ and PIWI domains were found in all 14 AGO sequences whereas presence of other conserved parts such as MID, N terminal and linker domains were not predicted in all candidates when applying default settings (Supplementary Fig. S2a). Based on sequence identity and phylogenetic clustering, the orthologs of *Arabidopsis AGO1, AGO2, AGO3, AGO4, AGO5, AGO6, AGO7* and *AGO10* were all discovered in the potato genome (Supplementary Fig. S2). In addition, two orthologs each for *AGO1, AGO2, AGO10* and three for *AGO4* were identified. *AGO6* was not found in the reference annotation of the potato genome (PGSC) but later identified in the annotation by the International Tomato Annotation Group (ITAG). Two candidates, *AGO8* and *AGO9* were however not found in any dataset. Phylogenetic clustering conferred five homologs of *StAGO*s in each clade of *AGO1* and *AGO4*, while four homologs grouped in the *AGO2* clade based on the AGO classification in *Arabidopsis*^19^. The *StAGO* paralogs showed varied degree of identity and genetic distances among each other (Supplementary Fig. S2b). Two candidates, *StAGO15* and *StAGO7* were the most divergent AGOs compared to the other members.

Next, the StAGOs were mapped on the *S. tuberosum* chromosomes (Supplementary Fig. S3). The close positions of *StAGO2a*, *StAGO2b* and *StAGO3* on chromosome 2, and *StAGO1a*, *StAGO4a* and *StAGO10c* on chromosome 6, together with sequence similarities, suggest that they have experienced gene duplications. Similar tandem gene duplications are observed on chromosome 2 and 6 in tomato^25^. In tomato and potato, no *AGO*s are found on chromosome 4, 5, 8, 10 and 11.

**Figure 2:**
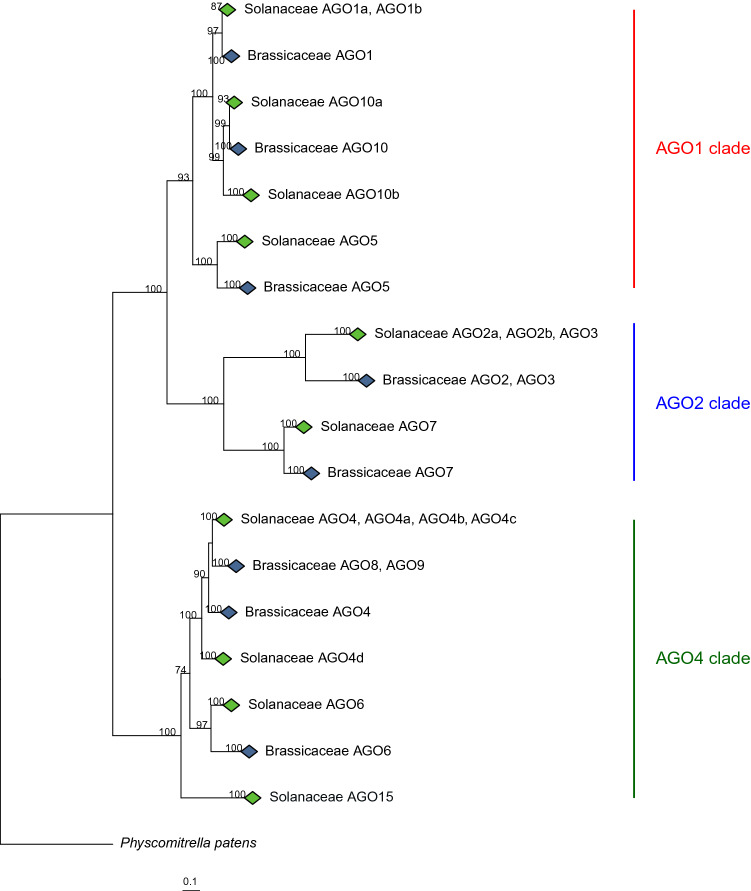
Maximum likelihood phylogeny (RAxML, model JTT + Γ, 100 replicates) of Argonaute (AGO) family in Solanaceae and Brassicaceae. Blue and green diamonds represent collapsed Solanaceae and Brassicaceae clades, respectively. Bootstrap values > 70% are indicated. Bar = number of substitutions per site. Outgroup = *Physcomitrella patens*.

A maximum likelihood phylogeny was reconstructed by using a total of 203 *AGO* homologs from the sampled Solanaceae lineages. To get confidence over the topology and partitions, 99 AGO homologs were added from nine sequenced Brassicaceae species (Supplementary Fig. S4). In line with earlier clustering (Supplementary Fig. S2b) three major clades (AGO1, AGO2, AGO4) were formed (Fig. 2). Homologs from almost all species were observed in all three clades including duplications in Solanaceae. The clustering pattern and topology indicates that a duplication of *AGO10* has occurred in an early ancestor prior the divergence of Solanaceae and Brassicaceae, but the duplicated ortholog has been lost in Brassicaceae. The duplication of the *AGO1* gene, on the other hand, has most likely occurred early at the base of Solanaceae after the split with Asterids. Two gene copies of *AGO1* and *AGO10* were found in *Nicotiana benthamiana,* and two copies for A*GO5* in the four species *Petunia inflata*, *N. tabacum*, *N. tomentosiformis* and *N. benthamiana*. Orthologs in the AGO5 sub-clade are more dissimilar than those in the AGO1 and AGO10 sub-clades, a situation which also is reflected in variations among the branch lengths. Orthologs of *AGO7* were observed in almost all Solanaceae lineages and a separate partition with the Brassicaceae orthologs formed the AGO7 sub-clade. Almost the same branch length indicates low variation among the orthologs. Further, *AGO2* and *AGO3* are sister orthologs that grouped together forming a separate sub-clade in which Solanaceae lineages showed varied number of paralogs (Fig. 2). Only one ortholog for *AGO2* and *AGO3* was detected from Nicotiana, Petunia and Capsicum lineages, while the Solanum species analyzed had three orthologs, except *S. chacoense* that had two gene copies.

The AGO4 clade was most likely formed by orthologs of *AGO4* exhibiting clear partition with AGO8 and AGO9 sequences from Solanaceae (Fig. 2). Two groups of *AGO4* orthologs were found in Solanaceae. Based on identity with the corresponding members in *Arabidopsis*, three paralogs in for example potato were found (*StAGO4*, *4a* and *4d*). Orthologs of *AGO6* from the two plant families were also identified.

### StAGO15 has novel domain architecture

**Figure 3:**
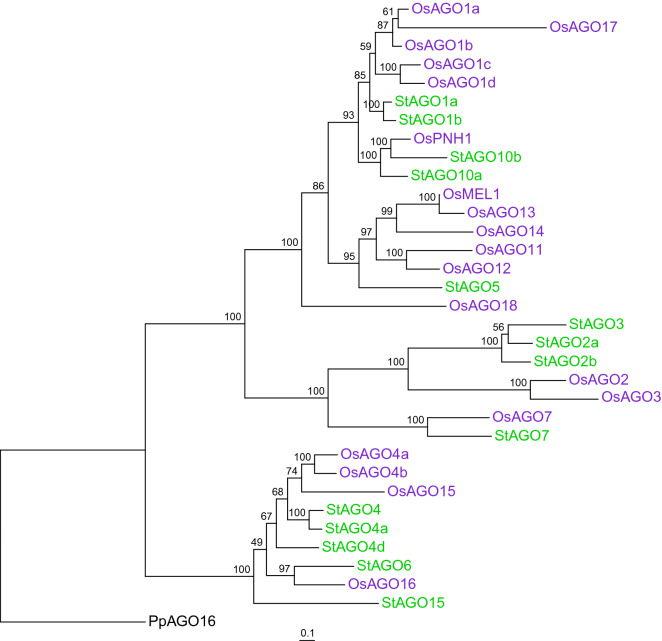
Maximum likelihood phylogeny (RAxML, model JTT + Γ, 150 replicates) of the Argonaute (AGO) family in potato (green) and rice (purple). Bootstrap values > 70% are indicated. Outgroup = *Physcomitrella patens* AGO16. Bar = number of substitutions per site.

A well supported sub-clade containing a single member from each Solanaceae species, except from *N. attenuata* and *N. benthamiana*, was found at the base of the AGO4 clade, earlier annotated as *AGO15* in tomato^25^ (Fig. 2; Supplementary Fig. S4). The relative position of this node in the phylogeny and branch lengths suggests that the evolution of AGO15 occurred early in Solanaceae. We searched databases and compared AGO15 sequences from Solanaceae and Poaceae in order to detect potential similarities in sequence and function. The phylogenetic tree generated two monophyletic clades with representatives from each plant family (Supplementary Fig. S5). The topology of the tree coincides with estimated divergence time between Monocots and Eudicots^26^. In rice, the AGO4 clade comprise of four members, *OsAGO4a*, OsAGO4b, *OsAGO15* and *OsAGO16* (Fig. 3). StAGO15 does not cluster with any of the potato or rice AGOs in this clade, suggesting independent evolution.

We aligned the AGO15 and the AGO1 clade sequences and found clear divergence in the PIWI domain among the Solanaceae species, particularly among the amino acids corresponding to the catalytic “slicing” residues D-E-D-H/D (Supplementary Fig. S6). In potato, StAGO15 has replaced the catalytic tetrad ‘D-E-D-H/D’ with a G-E-Q-R motif with unknown slicer function. Likewise, tomato has a G-Q-R/P motif at the catalytic site of SlAGO15^25^.

We closer examined the MID domains that did not fulfill the default sequences in comparison with AtAGO1 (Supplementary Fig. S7a,b). Of particular interest was the nucleotide specificity loop (NSL) which in *Arabidopsis* is known to regulate 5′ specificity (C, U or A)^9,10,27^. The Solanaceae AGO15 protein sequences deviate substantially from the AGO1 clade in the NSL positions, where for example StAGO15 has AFY as 5′ end recognition sequence (Supplementary Fig. S7b).

**Figure 4:**
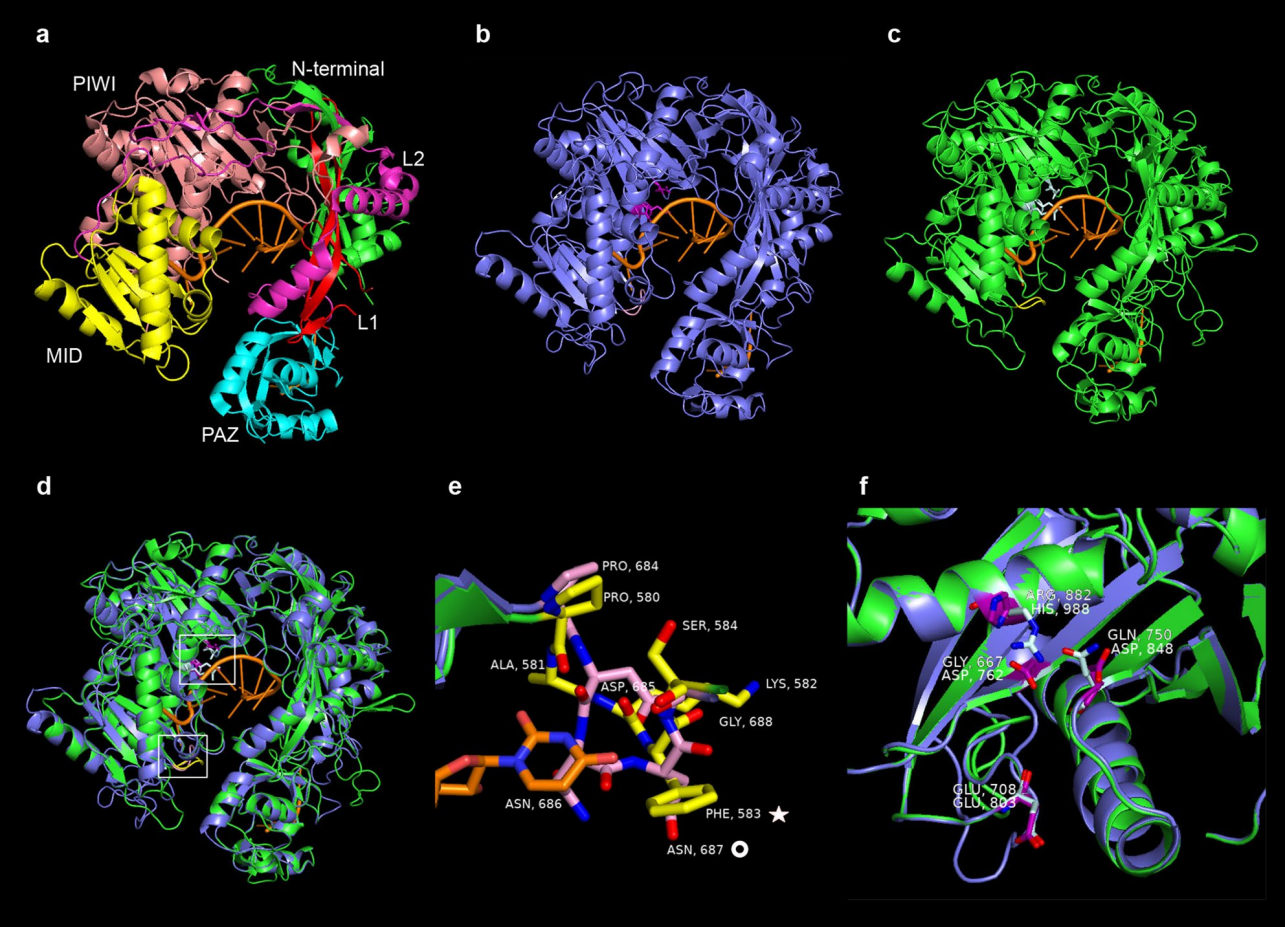
Three-dimensional AGO protein structure predictions. (**a**) Crystal structure of the human AGO2-miR-20a complex. N-terminal = green, L1 = red, PAZ = cyan, L2 = magenta, MID = yellow, PIWI = salmon. (**b**) AtAGO1 (steel blue) and Hs-miR20 (orange, PDB ID 4F3T:R). Residues of the D-E-D-H motif are in purple. The nucleotide specificity loop (NSL) in pink. (**c**) StAGO15 (green) and Hs-miR20 (orange). Residues of the G-E-Q-R motif in light cyan. The NSL in yellow. (**d**) Figure b and c merged. (**e**) Close view of the NSL in figure d (upper square). Nitrogen (red) and oxygen atoms (blue). The replacement of Asparagine (ASN, 687) in AtAGO1 to Phenylalanine (PHE, 583) in the StAGO15 NSL is highlighted with a white star (PHE, 583) and a white ring (ASN, 687). (**f**) Close view of the D-E-D-H/G-E-Q-R motives in figure d (lower square, Hs-miR20 removed). Nitrogen (red) and oxygen atoms (blue). StAGO15 residue labels are placed above the AtAGO1 labels.

A three-dimensional protein structure comparison was performed by first model the human AGO2 protein to visualize the different main domains together with their interaction with miR20a^28^ (Fig. 4a). Next, models of AtAGO1 and StAGO15 were constructed to facilitate identification of divergent units (Fig. 4b,c). Merged protein structures of AtAGO1 and StAGO15 showed large similarities (Fig. 4d). However, the StAGO15 protein appeared somewhat “bulky”. This feature is explained by three single coils, located either at the N-terminal, in the L1 domain or at the opening of the central pocket. In comparison with AtAGO1, the NSL sequence of StAGO15 has a hydrophobic residue (Phe583) replacing Asn 687 in AtAGO1 (Fig. 4e). This residue is of importance for the 5′ nucleotide selection in *Arabidopsis*^9^. Further, the D-E-D-H catalytic pocket observed in AtAGO1 is replaced by a G-E-Q-R motif in StAGO15 (Fig. 4f). The D-E-D-H and G-E-Q-R motifs resemble each other, sharing the glutamic acid (Glu 708 vs. Glu 803) as 2nd motif residue with negative charge and a 4th positive residue (Arg 882 vs. His 988). The 3rd motif residue, being negative in AtAGO1 (Asp 848) and polar in StAGO15 (Gln 750) is in both cases capable of binding positive residues, hence this substitute may not affect the function of StAGO15. The charge of Gly 667 in G-E-Q-R is pH dependent, a feature whose impact is unknown particularly under stress condition. The divergent recognition and binding motifs compared to AGO1 clade may suggest specific function(s).

### StAGO15 is elevated upon pathogen infection

**Figure 5:**
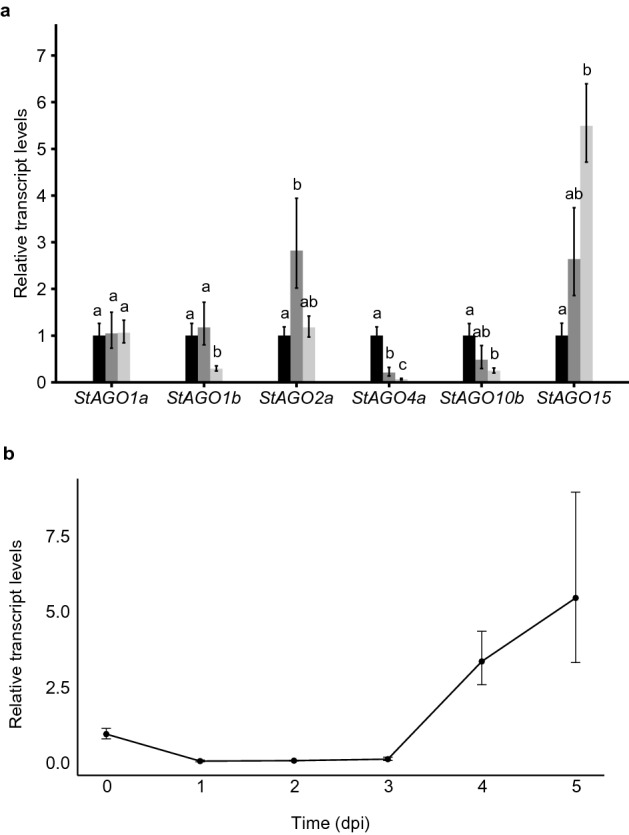
Argonaute expression in potato cv. Desirée during pathogen challenge. (**a**) Relative transcript levels of *StAGO* genes during pathogen challenge five days post infection (dpi). Black = H_2_O, dark grey = *A. solani*, light grey = *P. infestans* NL 11388 strain. Error bars indicate mean ± standard error of the mean (n = 4). Letters in the bar charts (a–c) represent significant differences (one-way ANOVA and Tukey’s HSD test: P < 0.05). (**b**) Relative transcript levels of *StAGO15* in a time-course from 0 to 5 dpi when infected by the *P. infestans* NL 11388 strain. Error bars indicate mean ± standard error of the mean (n = 4).

Based on generated RNAseq data on potato challenged by the *Phytophthora infestans* virulent strain 11388 *StAGO4c*, *StAGO10b*, and *StAGO15* were found up-regulated (Supplementary Fig. S8). Quantitative real-time PCR supported the activation of StAGO15 (Fig. 5a). In a time-course experiment the gene activation was clearly observed 4 to 5 days post inoculation (Fig. 5b, Supplementary Fig. S9) when *P. infestans* has switched from biotrophic to necrotrophic stage^29^. To clarify if this elevated A*GO15* activity was specific for *P. infestans,* the early blight fungus *Alternaria solani* was used for potato infection in parallel experiments. Again, *StAGO15* was up-regulated but not as much as seen in the *P. infestans* response (Fig. 5a).

## Discussion

The *Solanaceae* plant family comprises many important crop species with variable genome and gene family sizes, reflecting their history of genome duplications and variable selective constrains^22,30^. In an analysis of twelve Solanaceae species, gene duplication rate, strength of selection, and gene function was shown to vary extensively together impacting the gene family sizes^24^. Genes were detected enriched in the genomes either by whole genome duplication or by tandem duplication. Members in gene families with low domain variability displayed a tendency of housekeeping functions. Aforesaid genes appeared to have duplicated by whole genome duplication, in contrast to the tandem duplicated category that showed higher variability. In our analysis of *AGO* genes in 15 Solanaceae species a rather extensive variation in gene numbers were detected, particularly when comparing species in the Nicotiana genus with numbers in the genera Solanum and Petunia. In potato, remnants of *AGO* gene duplications can be observed on chromosome 2 and 6 and in tomato on chromosome 1, 2, 3, and 6^31^. In potato, we discovered three *StAGO4* (*4*, *4a*, *4d*) genes whereas four was found in tomato (*SlAGO4a, 4b, 4c, 4d*). When considering branch lengths, AGO4 is closer to AGO4b compared to AGO4c (Supplementary Fig. S3). In this case it is still not clear whether an incomplete gene duplication or a gene loss has occurred.

The split between Nicotiana and Solanum species is estimated to be rather recent *c*. 24 Myr^22^, hence gene family expansion and gene turnover rates should not deviate much between the two genera as found in our analysis. We can only speculate that human selection and clonal propagation could have had a major impact on gene content beside different duplication mechanisms as earlier suggested^31^.

It is believed that a functional RNAi pathway was present in the last common ancestor for eukaryotes as a defense system against viruses and transposons, a system that has expended to comprise regulation of endogenous RNAs^32^. Members of AGO proteins can be found in a majority of eukaryotic super-groups, where AGOs act as partners in a multi-protein regulatory system impacting an array of processes^15^. This multi-function feature also applies on plants, including defense. For example, AGO1, AGO2, AGO4, AGO5, AGO7, and AGO10 in *Arabidopsi*s are known to participate in mechanisms involving defense responses towards different types of viruses^33,34^. More precisely antiviral AGOs associate with virus-derived small RNAs to repress complementary viral RNAs or DNAs, or with endogenous small RNAs to regulate host gene expression and promote antiviral defense. In infected *N. benthamiana* plants, 21 and 22 nt sRNA from the potato spindle tuber viroid associate with AGO1, AGO2 and AGO3, while 24 nt viroid sRNA bind to AGO4, AGO5, and AGO6^35^. Similar events are also reported from rice where OsAGO1 and OsAGO18 act against *Rice stripe tenuivirus* and *Rice dwarf phytoreovirus*^36^. Not much is known about function of OsAGO15. It is believed to have evolved by duplication events followed by differentiation within the AGO4 clade^37^. OsAGO15 is expressed in reproductive tissue and harbor a D-D-H/P catalytic motif.

We checked for the presence of nuclear export signal (NES) and the nuclear localization signal (NLS) domains in the 14 potato AGO sequences. StAGO1a and 1b were the only AGOs containing both NES and NLS domains, known to be of importance for nuclear-cytoplasmic shuttling^38^. High scores of NLS were only detected for StAGO15, suggesting nuclear localization. For nuclear to cytoplasm transport, potato has five members in the exportin family. However, details on translocation from the nucleus, including loading partners and associated processes reported in *Arabidopsis*, are not known in potato. The protein sequence of StAGO15 differs at the NSL and the catalytic tetrad sequences compared to AtAGO1. The G-E-Q-R motif is so far only observed in the Solanaceae AGO15 proteins. Uracil is the most preferred 5′ nucleotide of AtAGO1 bound sRNA^10^, however adenine is the most hydrophobic nucleotide. The change from the polar Asn 687 in AtAGO1 to the hydrophobic Phe 583 in StAGO15, could indicate a preference for adenine as the 5′-nucleotide of sRNA binding. In *Arabidopsis* 5′ A is a signature for a loading preference of 24 nt sRNAs^39^. These features open up several functional possibilities, including induction of 24 nt phasiRNAs upon pathogen infection. Overlapping functions cannot be excluded at this stage. Resistance genes can become negatively regulated by host miRNAs upon pathogen attack as a self-defense response. In tomato, particularly the miR482/2118 family are active and *R* gene mRNA can be targeted both by these miRNAs and by self-generate secondary sRNAs^40^. There are many *R* genes in individual plant species, not least in potato, and it is thought that self-suppression by RNA silencing is a strategy to balance costs and benefits under pathogen attack. However, there is a complex co-evolutionary relationship between sequence diversity in *R* genes and interactions of evolving miRNA where much remains to be clarified^41^. In this context adds the potential impact of miR8788 from *P. infestans* on susceptibility in potato during infection another level of complexity^42^.

## Methods

### Dataset assembly

Genome sequences with annotated gene models present in Solanaceae databases (http://solanaceae.plantbiology.msu.edu/index.shtml, https://solgenomics.net) were searched to identify AGO homologs. A HMM-profile search using ‘HMMER’^43^ with *e* value of 0.0001 was applied on the translated version of in house transcriptome data, generated from potato cv. Sarpo Mira inoculated with *Phytophthora infestans*^44^. Only full length, characterized plant AGO sequences (either at amino acid or transcript level), containing at least three characteristic domains, were downloaded from UniProt^45^. *Arabidopsis* and rice AGO homologs were retrieved from TAIR; www.arabidopsis.org and RGAP; http://rice.plantbiology.msu.edu/. In total, 84 AGO protein sequences from 15 genomes and ten transcriptomes were used to construct the HMM profile. AGO sequences from Arabidopsis and tomato were used as queries for homology searches using tBLASTn^46^ with *e* value 0.001 to mine the genome sequences. The above methods were also used for the mining of AGO homologs in other species in the* Solanaceae* family. Next, potential homologs were further confirmed by the presence or absence of three characteristic domains: N terminal, PAZ and PIWI using Pfam^47^. Domains were predicted using HMMscan (HmmerWeb version 2.41.1). Default parameters (cut-off values: > 25 sequence and 22 hit bit scores) were applied^48^. Tandem duplicated gene pairs were identified if gene pairs were located within a distance of 100 kb on the genome or if the gene pairs were separated with four genes from each other.

### Phylogenetic analysis

The AGO homologs were aligned using ‘MAFFT v7.123b^49^ with 'ensi' option. Poorly aligned regions were cleaned using ‘trimAl’^50^ and option ‘Automated1’. Phylogenetic trees were reconstructed using Maximum Likelihood (ML) method as implemented in RAxML v 8.2.11^51^. The best substitution model JTT + Γ was applied for all trees. Robustness of the topologies and branches were assessed with 100 or 150 bootstrap replicates. The AGO homolog from *Physcomitrella patens* was used as outgroup for the rooting of the analysis and the R package ggtree for drawing. To infer evolutionary events, the AGO gene family tree was reconciled with the species tree, generated by the NCBI taxonomy browser, using NOTUNG^52^. *Erythranthe guttata* was used as outgroup in the gain and loss gene predictions. Pairwise identities, genetic distances and corresponding Neighbor-Joining tree were computed using MEGA v.7^53^.

### In silico protein analysis and modeling

Alignment of the MID domain and the D-E-D-H/D motif was displayed with the R package ggmsa and the secondary structure of the MID domain was predicted by the RaptorX-Property tool^54^. The potato MID domains are estimated from aligning them to the MID domain of AtAGO1^27^. Protein sequences of AtAGO1 and StAGO15 were used for three-dimensional structure modeling with SWISS-MODEL, against the AGO2-miR-20a complex (PDB ID 4F3T^28^), being the template with highest Global Model Quality Estimation number (0.61) and providing a miRNA to the model. The PyMOL Molecular Graphics System (PyMOL) was utilized for visualization of the predicted structures^55^. NES were predicted applying NESmapper (https://sourceforge.net/projects/nesmapper/) and NLS were predicted with cNLS Mapper (http://nls-mapper.iab.keio.ac.jp/cgi-bin/NLS_Mapper_form.cgi).

### Pathogens and inoculations

Potato plants (cv. Desirée) were inoculated with *Phytophthora infestans* strain NL11388 as earlier described^44^. Leaf inoculations using 10 µl of a 20 × 10^4^ spores/ml water of the fungus *Alternaria solani*, strain 142.2 collected from a field in Nymö, located in southern Sweden^56^ were also performed. Sterile water was used as control.

### Quantitative real time PCR

Total RNA was isolated from infected potato leaves and control samples using RNeasy Plant Mini Kit (Qiagen) according to the manufactory’s recommendation. Prior to qRT-PCR analysis, 1 µg dsDNase (Thermo Scientific) treated RNA was reverse-transcribed into cDNA using Maxima H Minus First Strand cDNA Synthesis Kit (Thermo Scientific). At least four biological replicates were examined using iTaq Universal SYBR Green Supermix (Bio-Rad).

Sequences of DNA oligonucleotides are listed in Supplementary Table S1. The *StEF1α* and *StACT101* gene were used as the internal reference^57^.

### *P. infestans* DNA quantification

To evaluate *P. infestans* infection, its genomic DNA (gDNA) was quantified by qPCR essentially as described earlier^42,58^. Genomic DNA was isolated from potato leaves inoculated with *P. infestans*. Concentration of obtained gDNA was determined using Qubit dsDNA BR Assay Kit (Thermo Scientific). qPCR analyses with four biological replicates were carried out using iTaq Universal SYBR Green Supermix (Bio-Rad). 20 ng gDNA was used as template in each qPCR reaction together with primers for *PiO8* or *StACT101*. Primers are listed in Supplementary Table S1. All statistics were calculated as detailed as earlier described^42^.

### Transcriptome sequencing and bioinformatic analysis

Leaves were collected from potato plants (cv. Sarpo Mira) 5 days post inoculation (dpi) using *P. infestans* isolates 88069 and 11388. Uninfected and water inoculated leaves were used as controls. For each sample at least 3 leaves were pooled. Total RNA was extracted using the RNeasy mini kit (QIAGEN). Thirteen transcript libraries followed by Illumina HiSeq 2500 sequencing were performed at the National Genomics Infrastructure, Illumina sequencing platform (Stockholm). The Illumina adaptor sequences and low-quality reads were removed using Trimmomatic v0.36^59^. The filtered datasets were mapped to *S. tuberosum* v4.04 and the *P. infestans* reference genomes^60,61^ using kallisto v0.43.0^62^. Differential expression analysis was performed using the DESeq2 package^63^. All calculations were performed in R v3.2.0 (www.R-project.org).

## Supplementary information

Supplementary Information.

## Data Availability

Transcriptome data are available through the National Center for Biotechnology Information (NCBI) Gene Expression Omnibus, Series accession number GSE159015 (http://www.ncbi.nlm.nih.gov/geo/query/acc.cgi?acc=GSE159015). Additional sequence and phylogeny data can be found in the Treebase repository, http://purl.org/phylo/treebase/phylows/study/TB2:S26565.
