# Author Correction: Genome-wide identification of Argonautes in Solanaceae with emphasis on potato

**DOI:** 10.1038/s41598-021-91689-z

**Published:** 2021-06-10

**Authors:** Zhen Liao, Kristian Persson Hodén, Ravi Kumar Singh, Christina Dixelius

**Affiliations:** 1 Department of Plant Biology, Uppsala BioCenter, Linnéan Center for Plant Biology, Swedish University of Agricultural Sciences, P.O. Box 7080, 75007 Uppsala, Sweden; 2 Present Address: Genomics and Bioinformatics Laboratory, University Department of Botany, Magadh University, Bodh Gaya, 824234 India

Correction to: *Scientific Reports* 10.1038/s41598-020-77593-y, published online 25 November 2020

This Article contains errors in the Results section under subheading ‘Potato has 14 Argonaute encoding genes’.

“Next, the *StAGOs* were mapped on the *S. tuberosum* chromosomes (Supplementary Fig. S3). The close positions of *StAGO2a*, *StAGO2b* and *StAGO3* on chromosome 2, and *StAGO1a*, *StAGO4a* and *StAGO10c* on chromosome 6, together with sequence similarities, suggest that they have experienced gene duplications. Similar tandem gene duplications are observed on chromosome 2 and 6 in tomato^25^. In tomato and potato, no *AGOs* are found on chromosome 4, 5, 8, 10 and 11.”

should read:

“Next, the *StAGOs* were mapped on the *S. tuberosum* chromosomes (Supplementary Fig. S3). The close positions of *StAGO2a*, *StAGO2b* and *StAGO3* on chromosome 2, together with sequence similarities, suggest that they have experienced gene duplications. Similar tandem gene duplication is observed on chromosome 2 in tomato^25^. In tomato and potato, no *AGOs* are found on chromosome 4, 5, 8, 10 and 11.”

