# Supplementary material for: Genome-wide identification of Argonautes in Solanaceae with emphasis on potato
Source: Sci Rep. 2020 Nov 25;10:20577. doi: 10.1038/s41598-020-77593-y (PMC7689493; doi:10.1038/s41598-020-77593-y)
Supplement: Supplementary file 1 — Supplementary Information. [file 41598_2020_77593_MOESM1_ESM.docx]

**Supplementary Information**

**Genome-wide identification of Argonautes in Solanaceae with emphasis on potato**

Zhen Liao^†^, Kristian Persson Hodén^†^, Ravi Kumar Singh and Christina Dixelius

† = Joint first authors

**Supplementary Figures S1-S9**

**Supplementary Table S1**

**Supplementary Figures**

**
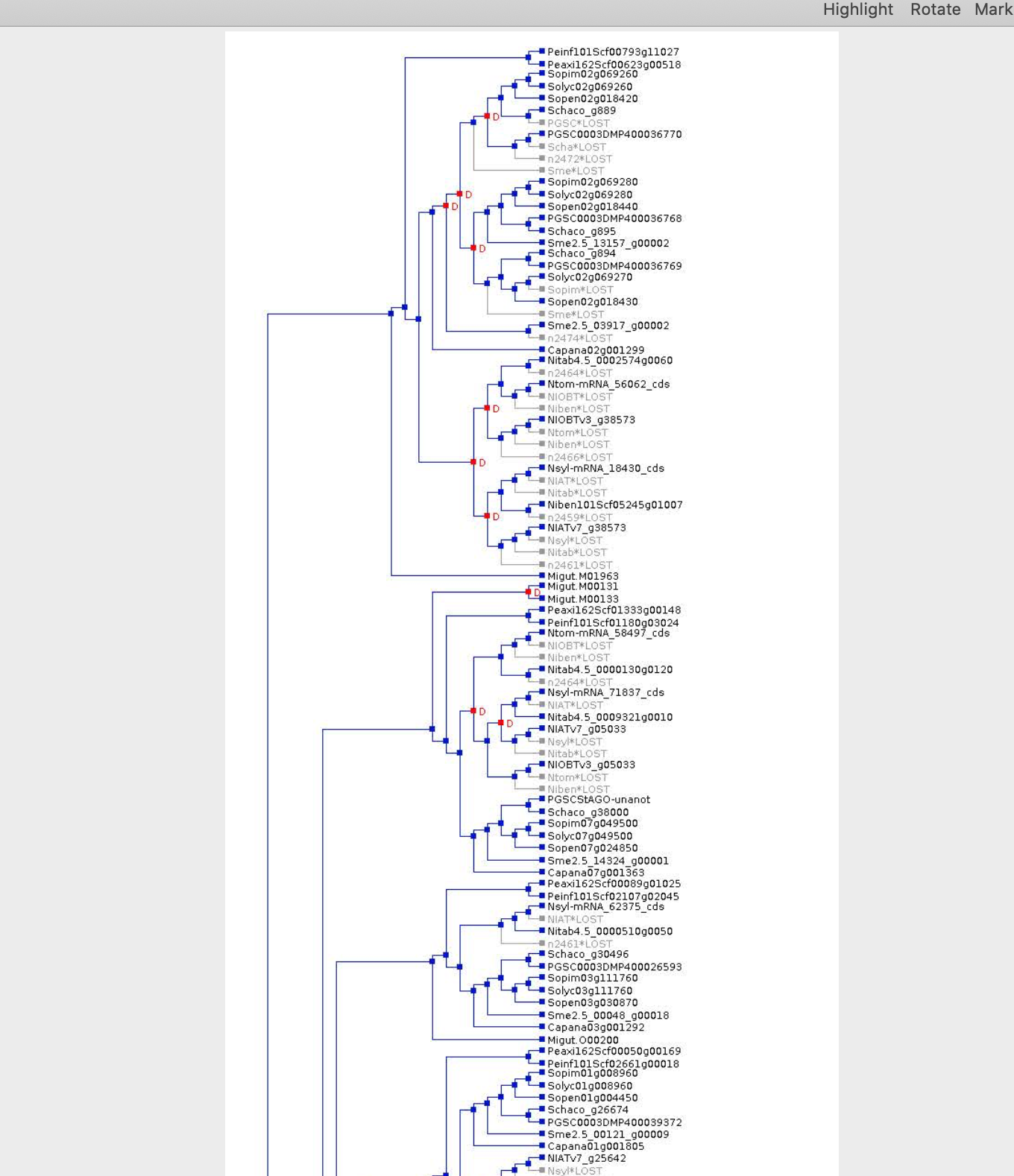
**

**Continue next page**

**
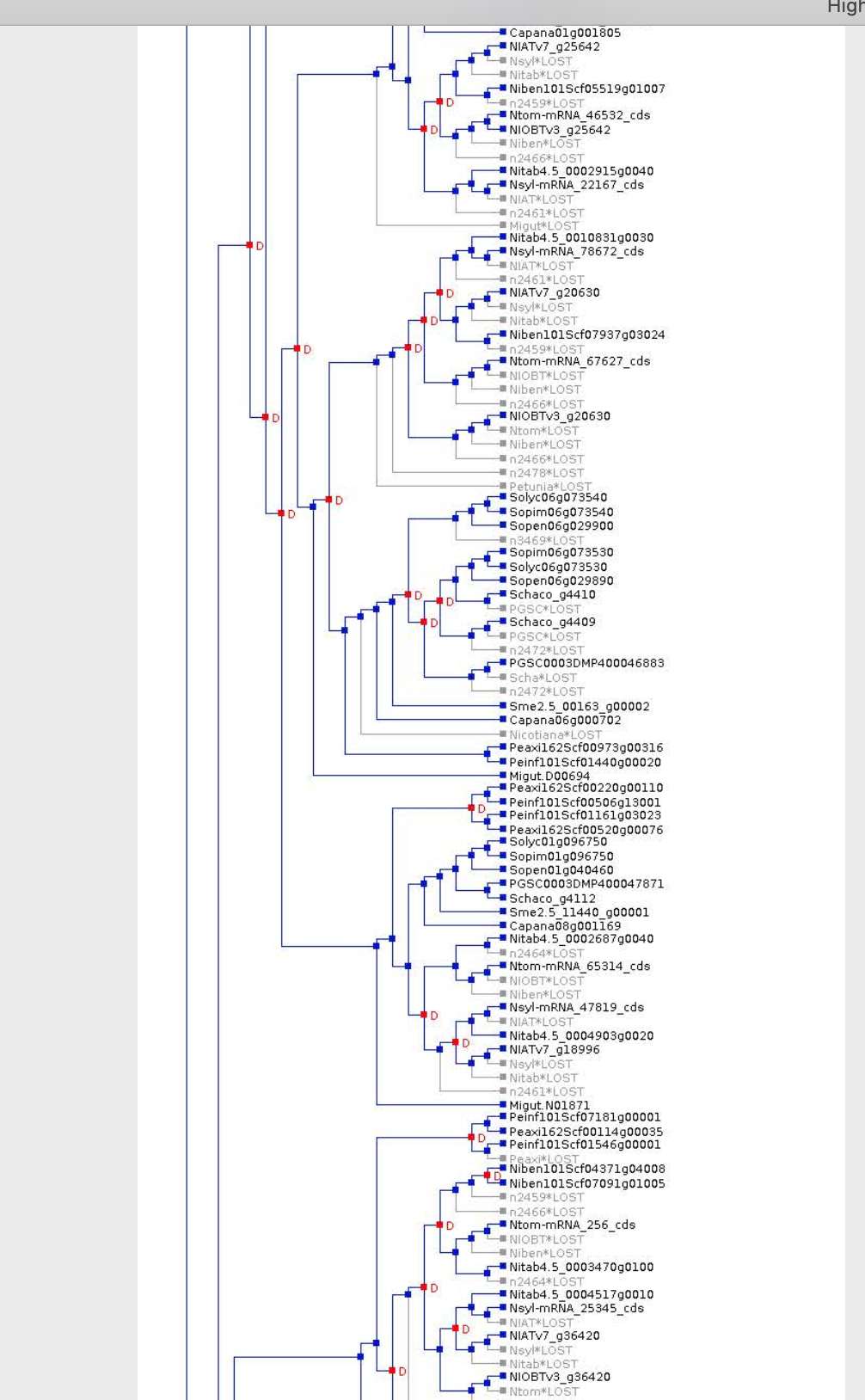
**

**Continue next page**

**
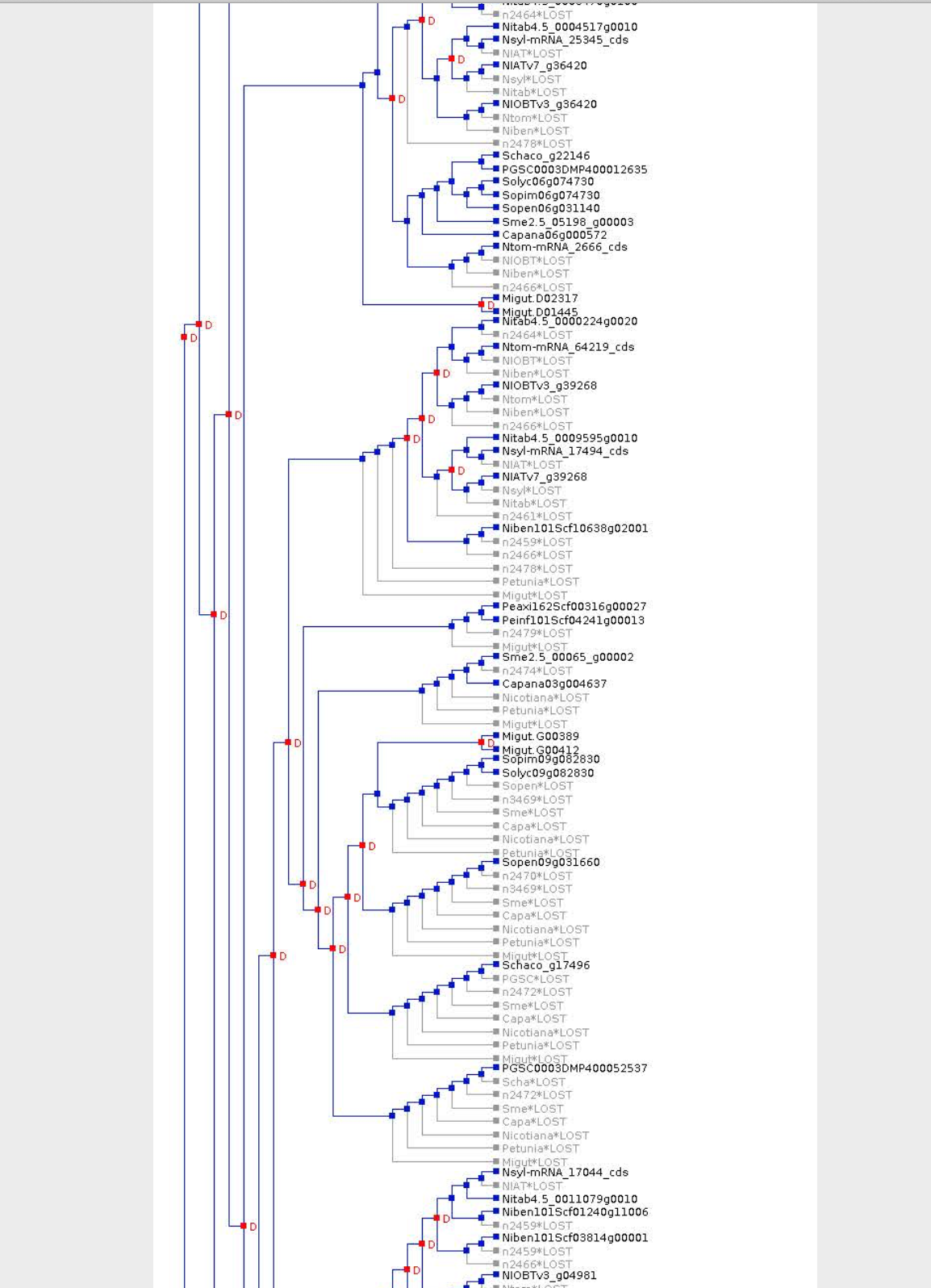
**

**Continue next page**

**
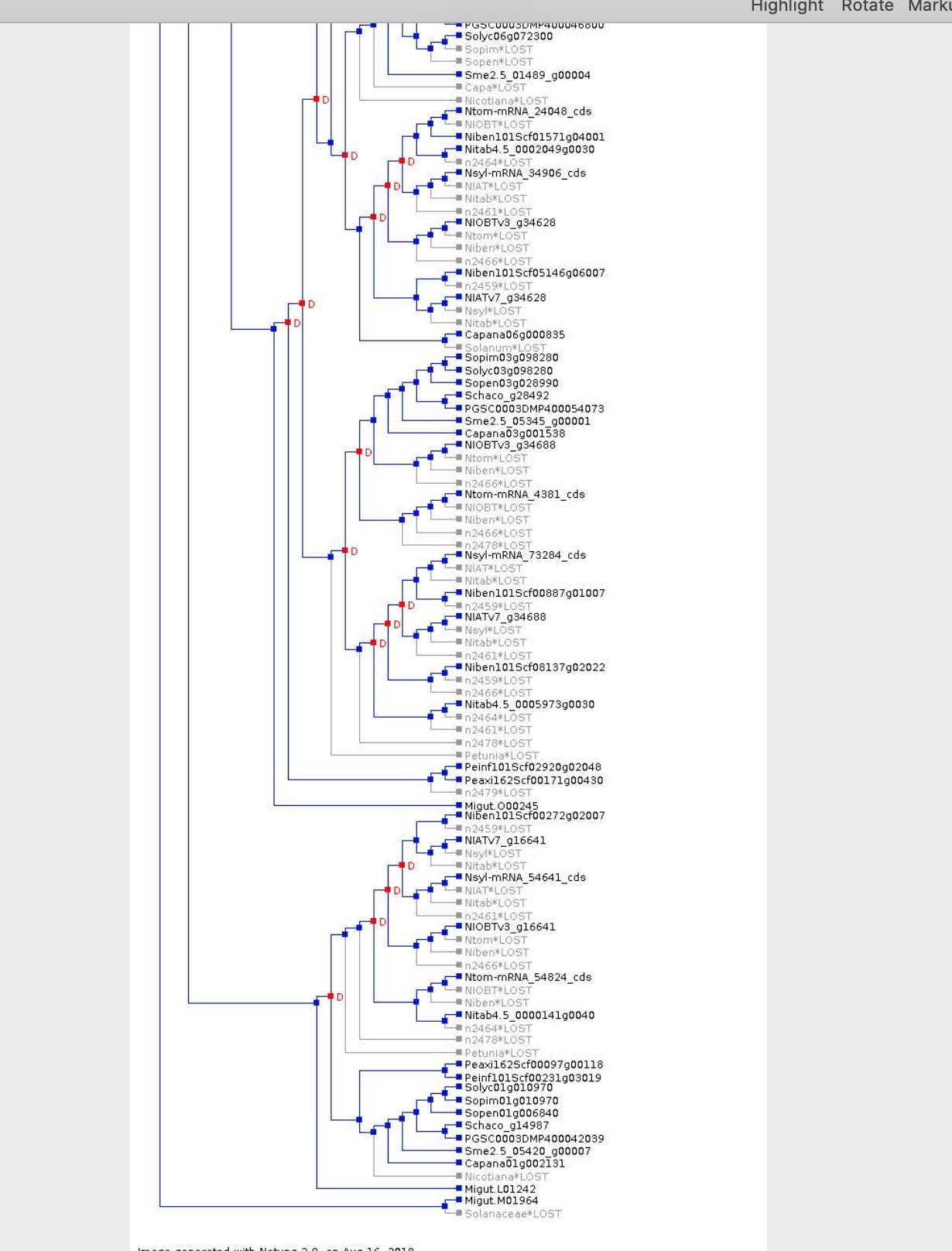
**

**Supplementary Fig. S1.** Reconciliated tree of AGO phylogeny showing duplication (D) events and gene losses during evolution of Solanaceae.


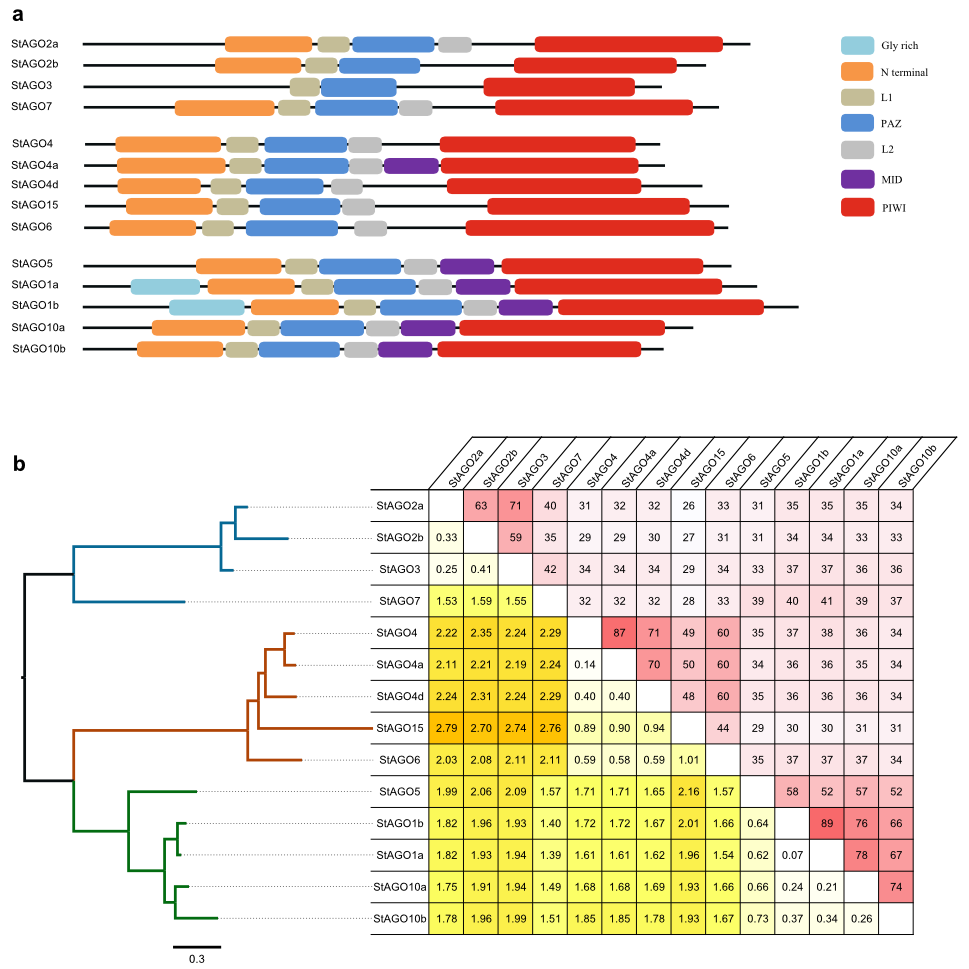


**Supplementary Fig. 2.** Characterization of *AGO* genes in the *Solanum tuberosum.* (a) Domain distribution in the StAGO proteins. Illustration not in scale. Domains were predicted using HMMscan (HmmerWeb version 2.41.1. Cut-off values = sequence and hit bit score of > 25 and 22, respectively (default parameters). (b) Neighbor-joining phylogenetic clustering and the distances (upper diagonal (red): pairwise identities; lower diagonal (yellow): genetic distances among the StAGO proteins.


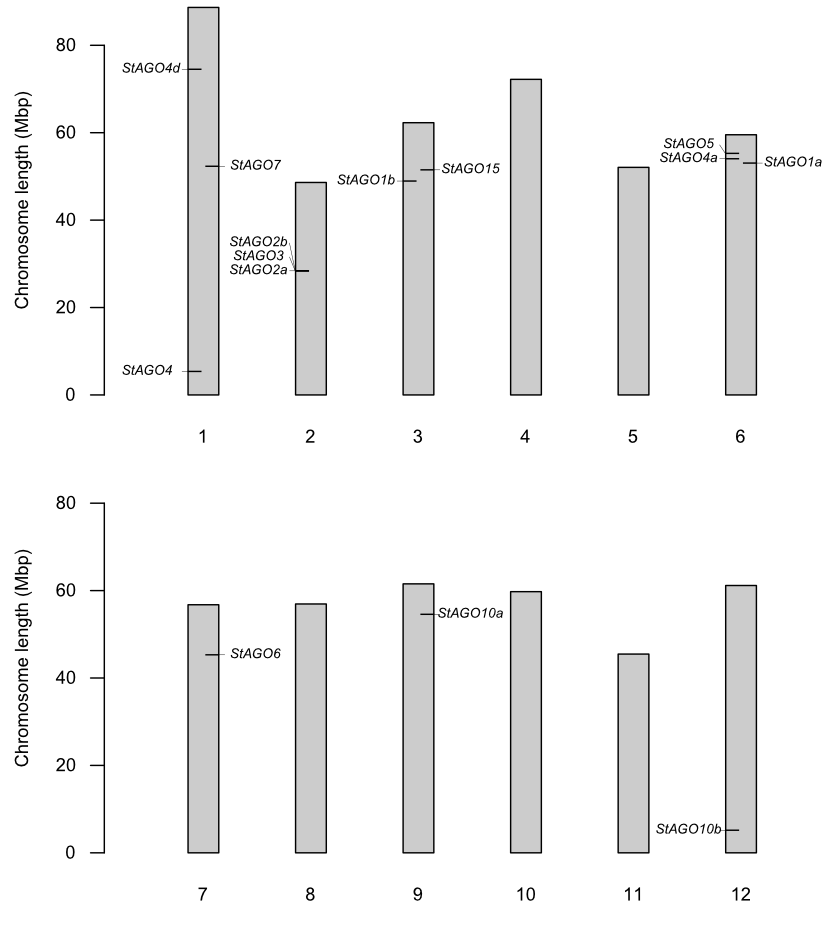


**Supplementary Fig. S3.** Distribution of *AGO* genes on the 12 potato chromosomes. Left side = positive strand, right side = negative strand.

**
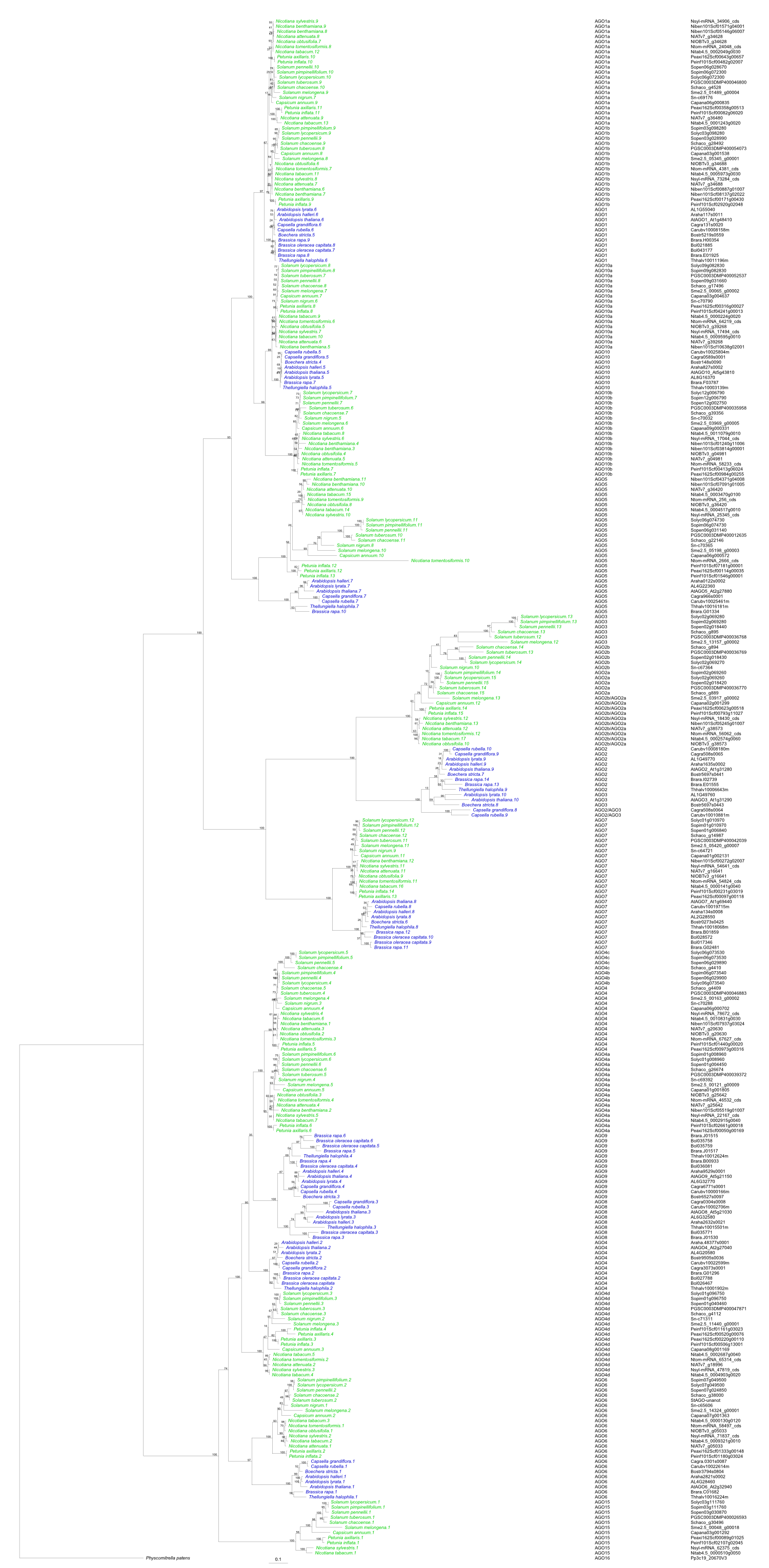
**

**Supplementary Fig. S4** Phylogenetic tree of the unrooted maximum likelihood phylogeny (RAxML, model JTT +Γ, 100 replicates) of Argonaute (AGO) family in Solanaceae (*Capsicum annuum,* *Solanum melongena*, *S. tuberosum*, *S. chacoense*, *S. pennellii*, *S. pimpinellifolium*, *S. lycopersicum*, *S. nigrum,* *Nicotiana tabacum*, *N. attenuata*, *N. sylvestris*, *N*. *benthamiana*, *N. obtusifolia*, *N. tomentosiformis*, *Petunia inflata*, *P. axillaris*) and Brassicaceae (*Arabidopsis thaliana*, *A. lyrata*, *A. halleri, Boechera stricta, Brassica oleracea*, *B. rapa*, *Capsella grandiflora*, *C. rubella*, *Thellungiella halophila*).

Solanaceae species are green and Brassicaceae species are blue. Bootstrap values > 70% are indicated. Outgroup = *Physcomitrella patens*. Bar = number of substitutions per site.


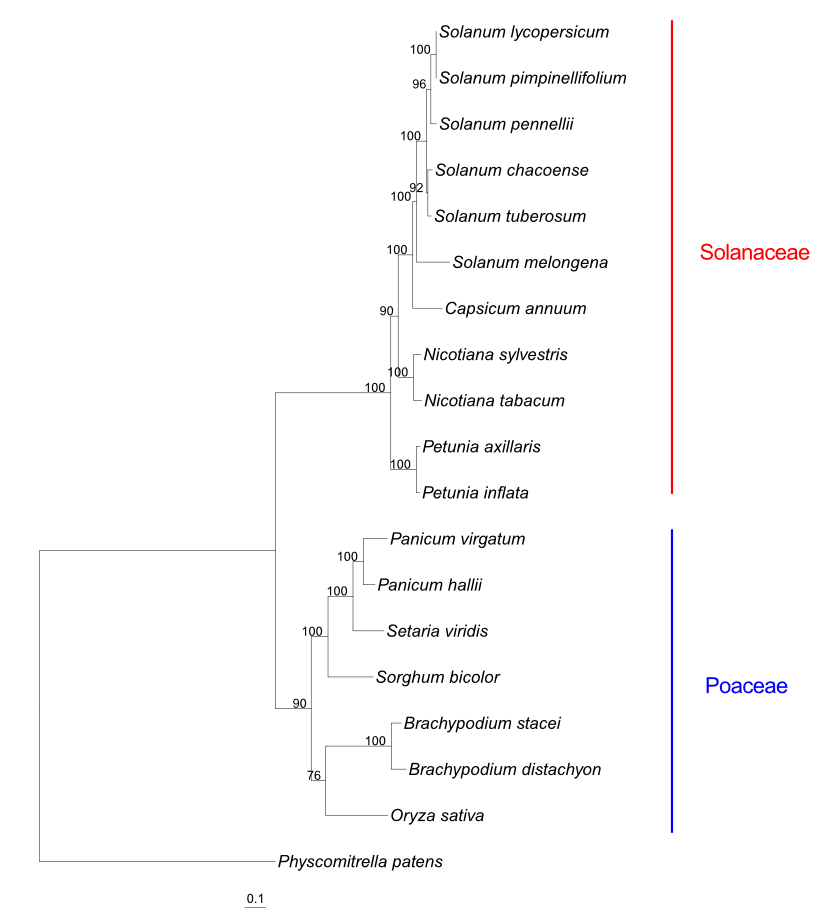


**Supplementary Fg. S5**. Phylogenetic tree of the rooted maximum likelihood phylogeny (RAxML, model JTT +Γ, 150 replictes) of AGO15 in Solanaceae and Poaceae. Bootstrap values > 70% are indicated. Outgroup = *Physcomitrella patens*. Bar = number of substitutions per site.


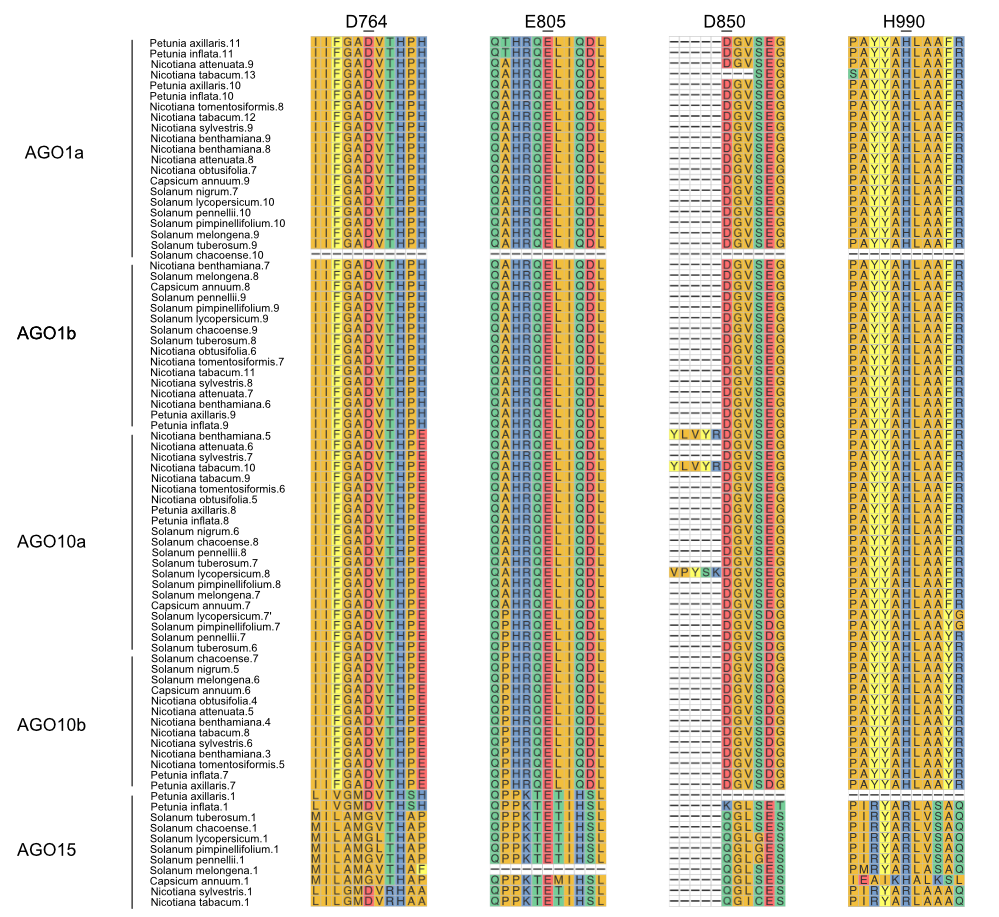


**Fig. S6** Alignment of the *Solanaceae* AGO1 clade and AGO15 sequences. The DEDH motif from the PIWI domain is displayed. Positions annotated are referring to positions of the StAgo1a sequence. The amino acids are coloured according to their chemical properties of their side chains (red = negative, blue = positive, green = polar uncharged, yellow = aromatic, orange = hydrophobic).


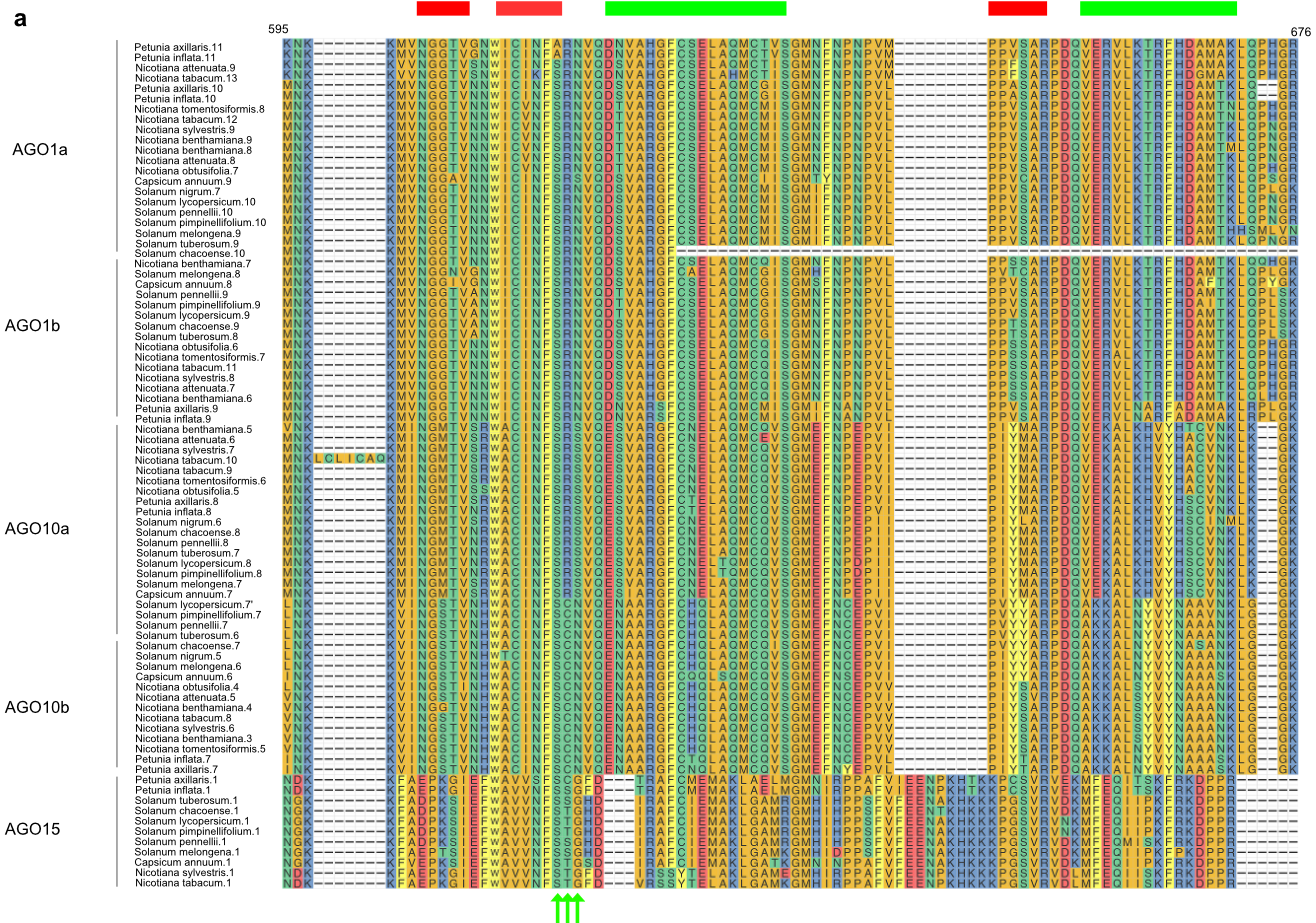


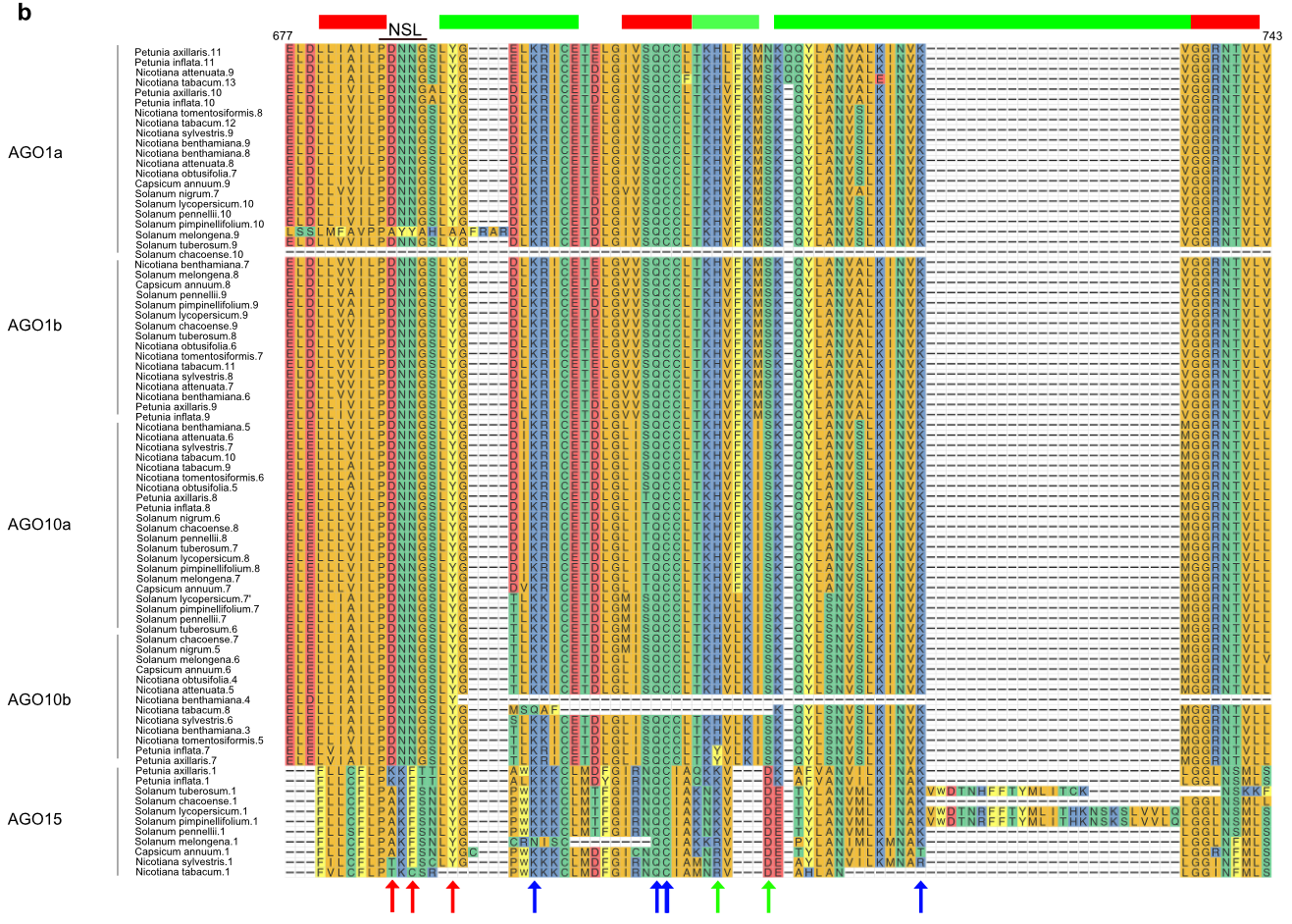


**Fig. S7.** Alignment of the MID domain in the AGO1 clade and AGO15 sequences in *Solanaceae,* divided in two sections. Positions annotated are referring to positions of the StAGO1a sequence. (a) Position 595-676. (b) Position 677-743 including the nucleotide specificity loop (NSL), encoded by the amino acids PAKFS in StAGO15. Color code = chemical properties of amino acid side chains (red = negative, blue = positive, green = polar uncharged, yellow = aromatic, orange = hydrophobic). Lines above the alignments represents secondary structure predictions (red = α-helix, green = β-sheet).

Arrows symbolize predicted functional residues in StAGO15: 5’-end nucleotide specificity recognition (AFY, red), 5’-end nucleotide phosphate binding (KQCK, blue) and sulfur binding (SSGK, green).


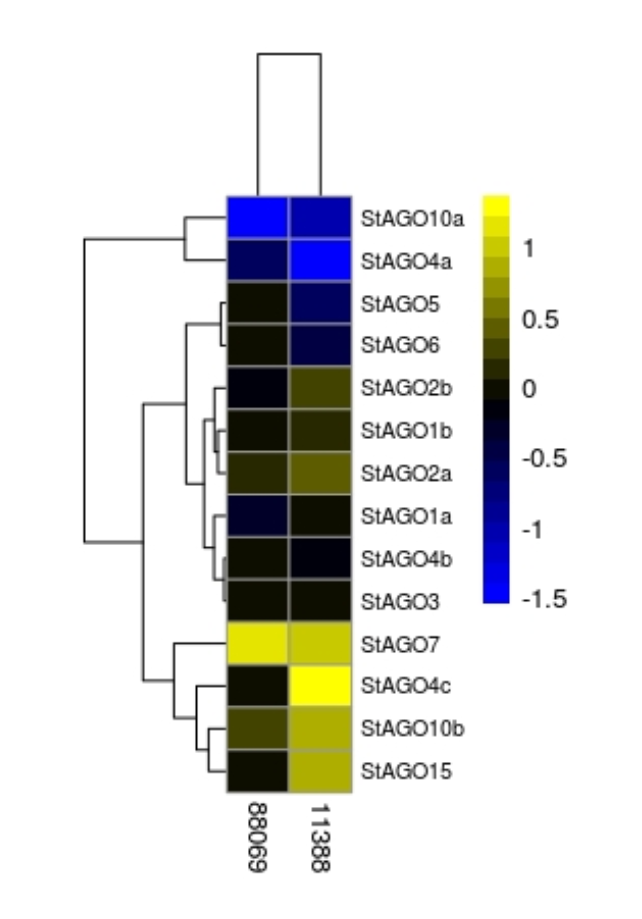


**Fig. S8**. RNA seq analysis of infected potato leaves (cv. Sarpo Mira), here visualizing the Argonaute responses 5 days post infection of *Phytophthora infestans* strain 88069 and the highly aggressive 11388 strain.


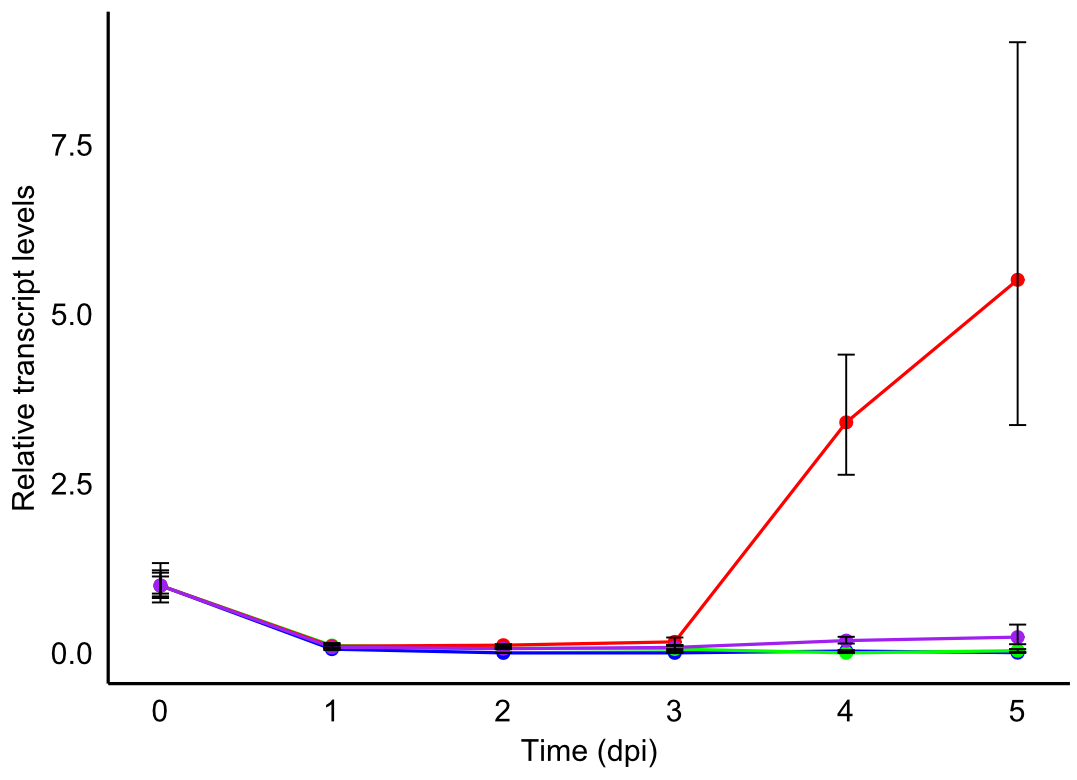


**Fig. S9**. Relative transcript levels of *StAGO1a* (purple), *StAGO10a* (blue), *StAGO10b* (green) and S*tAGO15* (red) in a time-course from 0 to five days post infection (dpi).

Potato cv. Desirée was infected by *P. infestans* NL 11388 strain. Error bars indicate mean ± standard error of the mean, (n = 4).

**Supplementary Tables**

**Table S1.** Primer sequences

| **Name** | **Sequence 5'-3'** | **Purpose** | **Target gene** |
| --- | --- | --- | --- |
| StACTIN_qF | GCCTCCTGAACGGAAGTACA | g.DNA qPCR | *StACT101* |
| StACTIN_qR | AATGGAAGGACCGGATTCAT | g.DNA qPCR, | *StACT101* |
| PiO8-R | GCCTTCCTGCCCTCAAGAAC | g.DNA qPCR | *PiO8* |
| PiO8-F | CAATTCGCCACCTTCTTCGA | g.DNA qPCR | *PiO8* |
| St-EF1a_qF | GATGGTCAGACCCGTGAACA | qRT-PCR | *StEF1α* |
| St-EF1a_qR | CCTTGGAGTACTTCGGGGTG | qRT-PCR | *StEF1α* |
| StAGO10a_qF | AAGGGGAAGAGGAGGCAGAA | qRT-PCR | *StAGO10a* |
| StAGO10a_qR | AAGGAAACCCTGAGCTACTTCC | qRT-PCR | *StAGO10a* |
| StAGO10b_qF | GCGTAGTAGAATGGAAGAAGTCAAG | qRT-PCR | *StAGO10b* |
| StAGO10b_qR | GCAGGGGAAACCTCAGAGTTATT | qRT-PCR | *StAGO10b* |
| StAGO15_qF | CACCACCTCCCAAAGAAATTCC | qRT-PCR | *StAGO15* |
| StAGO15_qR | ATCTTCTTTCCCTTGACCCCC | qRT-PCR | *StAGO15* |
| StAGO1a_qF | CCACCTGAACCAATGACACATC | qRT-PCR | *StAGO1a* |
| StAGO1a_qR | AGTGCCATTACTACCCTTTCCA | qRT-PCR | *StAGO1a* |
| StAGO1b_qF | AGCTGTAATGACTACTCAGCCC | qRT-PCR | *StAGO1b* |
| StAGO1b_qR | TGTCACTTGCAGAGTTGACATC | qRT-PCR | *StAGO1b* |
| StAGO4a_qF | GATTTGACAAGACTTGGAGAGACT | qRT-PCR | *StAGO4a* |
| StAGO4a_qR | GAGAAGAAACTTCGGGGCAC | qRT-PCR | *StAGO4a* |
| StAGO2a_qF | GTACCAGCAAGCCAACTCAC | qRT-PCR | *StAGO2a* |
| StAGO2a_qR | CTGGTGGAACAAGTGAAACCG | qRT-PCR | *StAGO2a* |
